# Maximizing Public Benefit From Opioid Settlement Resources

**DOI:** 10.1111/1468-0009.12450

**Published:** 2020-02-20

**Authors:** ROBERT P. PACK, CHERYL G. HEALTON, SANDRO GALEA

**Affiliations:** ^1^ East Tennessee State University College of Public Health; ^2^ ASPPH Task Force on Public Health Initiatives to Address the Opioid Crisis; ^3^ New York University College of Global Public Health; ^4^ Boston University School of Public Health, ASPPH Board of Directors

The historic tobacco master settlement agreement (MSA) between 46 State Attorneys General and the tobacco industry in 1999 had a range of consequences. It resulted in the closure of tobacco industry policy groups that undermined public health, sharply reduced tobacco marketing using cartoon characters (eg, Joe Camel) and paid product placement in television, film and other media, and created a new nonprofit foundation whose primary goal was to educate youth and prevent them from initiating tobacco use. It also resulted in more than $206 billion in resources being allocated to states, subject to appropriation by state legislators and governors. This enabled states to recoup the cost of medical and other treatment expenditures for tobacco‐related illness.^1^ However, by 2018, only 2.6% of the $206 billion in settlement and tobacco state taxes had been used for tobacco‐related harm mitigation or prevention.^2^

In 2018, Dr. Robert Redfield, director of the US Centers for Disease Control and Prevention called the current crisis of overdose deaths “the defining epidemic of our time.” The parallels between the opioid crisis and the historic tobacco crisis are striking: both ran rampant for decades before systematic legal and policy efforts to combat them took hold, both relied on addictive agents difficult to quit once tolerance is high, and both were driven by corporations that made billions of dollars from their sale, facilitated by aggressive, sophisticated, and targeted marketing.

There is now the potential for a settlement agreement between the plaintiffs and defendants in the myriad opioid lawsuits that are making their way through the federal court system. More than 2,400 municipalities, counties, and states have sued opioid manufacturers, pharmaceutical distributors, and corporate pharmacy chains for damages resulting from the opioid crisis. In contrast with the tobacco settlement, the primary basis for the opioid lawsuits is to secure damages resulting from the public nuisance created by the defendants and to mitigate the harm, rather than to recoup medical and treatment costs. Hence, Attorneys General and other plaintiffs in these lawsuits have the opportunity to negotiate settlement terms that assure funds will be used for maximum public health benefit relative to the opioid crisis.

Preliminary settlement discussions that have not included all parties total at least $48 billion,^3^ but experts estimate that the settlement could be in the range of $50‐100 billion.^4^ This pales in comparison to the actual cost of the nonmedical use of opioids, estimated to have caused at least $631 billion in economic damages in the past four years alone.^5^

Resources obtained from a potential settlement should be used exclusively for proven public health approaches that directly address the crisis. The key questions to ask at this point are: what should be done (ie, what programs, systems, policies, and infrastructure should be put in place or bolstered); to what extent should each be done; and how should the resources be allocated to execute such a plan?

## What Should Be Done?

The epidemic is expected to continue unless there is a large‐scale systematic implementation of evidence‐based programs and policies to combat it. The Association of Schools and Programs of Public Health (ASPPH) has released a report of the Task Force on Public Health Initiatives to Address the Opioid Epidemic, which outlines proven public health strategies to reduce risk for addiction and to mitigate further harm from the crisis.^6^ The Task Force made recommendations in the areas of substance use disorder prevention, harm reduction, access to medications for opioid use disorder, stigma reduction, advocacy, and changes to industry practices, but was silent on the proportion of total resources to invest in each domain.

In a 2018 *New York Times* article, 30 experts were polled regarding proportionate investments of a fictional $100 billion to address potential solutions to the opioid crisis.^7^ The panel recommended that 47% of resources go for treatment (including medications for opioid use disorder, Medicaid expansion, treatment for the incarcerated, and pre‐trial diversion), 27% for demand reduction (including preventive measures such as community development, post‐incarceration support, education, and pain research), 15% for harm reduction (including naloxone, syringe service and surveillance programs, supervised consumption spaces, HIV/hepatitis treatment, and drug checking, such as fentanyl test strips), and 11% for supply reduction (including prescription monitoring, local police, interdiction, and other diversion controls).

Although the results of this modified Delphi‐panel survey might reflect a number of biases, the poll may be a good starting point for conversation about resource allocation that could lead to more rigorous surveys involving a larger number of stakeholders asked a similar question with discrete response categories limited to evidence‐based approaches with proven returns on investment.

## How Should the Resources Be Distributed?

We believe that all resources from a potential opioid MSA should be used to address the addiction and overdose crisis and not be allocated to states for redirected appropriation. Attorneys General and judges must work diligently to avoid redirected appropriation for the obvious reason that it would be “penny wise and pound foolish” in light of the extraordinary scope of the opioid epidemic.

We propose a model that builds on public comments made by Pennsylvania Attorney General Josh Shapiro in November 2019, as reported by the Pittsburgh Post‐Gazette:^3^

- 15% should be dispersed to states proportionate to the burden of overdose mortality since 1999, to be used for their state‐based systems of medical care, workers compensation, and other state‐level costs, which could include economic impacts and labor force participation. The funds could be dependent on a state match to leverage state and local resources toward the same aim.
- 15% should be dispersed to cities and counties involved in the litigation proportionate to the burden of disease since 1999, to be used to bolster nonprofit health systems, mental health services agencies, social services organizations, community coalitions, and to support access to medications for opioid use disorder (MOUD) and other locally relevant treatment and recovery services. A local match might generate more resources to buttress the MSA‐derived funding.
- 70% should be used to create a national nonprofit organization to allocate resources rapidly and fairly to communities highly impacted by the opioid crisis and to mount efforts to prevent new areas from becoming afflicted. A feature of this organization should be a large‐scale treatment assurance program that guarantees provision of evidence‐based medical treatment, counseling, and recovery support services where needed most. The program could be modeled on the successful Ryan White HIV/AIDS program, with the federal government also using appropriate funds for the federal component of Medicaid and Medicare as well as funds for training, special pediatric efforts, and community‐based prevention programs in parallel.

To date, the plaintiffs have borne the cost of both the litigation and the crisis, and we recognize that the ideas presented here may be controversial. However, a new national nonprofit with a public sector board, together with increased federal and local support, would supply high‐need communities with fiscal and programmatic resources in a rapid systematic response using proven dissemination, implementation, and social franchising intervention tools to assure institutionalization of the programs and continuous quality improvement of such programs. This would provide, in our opinion, the best possible way to ensure high return on investment of settlement funds to mitigate the opioid epidemic.
